# Systems analysis of methylerythritol-phosphate pathway flux in *E. coli*: insights into the role of oxidative stress and the validity of lycopene as an isoprenoid reporter metabolite

**DOI:** 10.1186/s12934-015-0381-7

**Published:** 2015-11-26

**Authors:** Mareike Bongers, Panagiotis K. Chrysanthopoulos, James B. Y. H. Behrendorff, Mark P. Hodson, Claudia E. Vickers, Lars K. Nielsen

**Affiliations:** Australian Institute for Bioengineering and Nanotechnology (AIBN), The University of Queensland, St. Lucia, QLD 4072 Australia; Metabolomics Australia (Queensland Node), The University of Queensland, St. Lucia, QLD 4072 Australia

**Keywords:** Isoprenoids, Lycopene, Methylerythritol phosphate pathway, RpoS, ROS, *Escherichia coli*, Oxidative stress

## Abstract

**Background:**

High-throughput screening methods assume that the output measured is representative of changes in metabolic flux toward the desired product and is not affected by secondary phenotypes. However, metabolic engineering can result in unintended phenotypes that may go unnoticed in initial screening. The red pigment lycopene, a carotenoid with antioxidant properties, has been used as a reporter of isoprenoid pathway flux in metabolic engineering for over a decade. Lycopene production is known to vary between wild-type *Escherichia coli* hosts, but the reasons behind this variation have never been fully elucidated.

**Results:**

In an examination of six *E. coli* strains we observed that strains also differ in their capacity for increased lycopene production in response to metabolic engineering. A combination of genetic complementation, quantitative SWATH proteomics, and biochemical analysis in closely-related strains was used to examine the mechanistic reasons for variation in lycopene accumulation. This study revealed that *rpoS*, a gene previously identified in lycopene production association studies, exerts its effect on lycopene accumulation not through modulation of pathway flux, but through alteration of cellular oxidative status. Specifically, absence of *rpoS* results in increased accumulation of reactive oxygen species during late log and stationary phases. This change in cellular redox has no effect on isoprenoid pathway flux, despite the presence of oxygen-sensitive iron-sulphur cluster enzymes and the heavy redox requirements of the methylerythritol phosphate pathway. Instead, decreased cellular lycopene in the Δ*rpoS* strain is caused by degradation of lycopene in the presence of excess reactive oxygen species.

**Conclusions:**

Our results demonstrate that lycopene is not a reliable indicator of isoprenoid pathway flux in the presence of oxidative stress, and suggest that caution should be exercised when using lycopene as a screening tool in genome-wide metabolic engineering studies. More extensive use of systems biology for strain analysis will help elucidate such unpredictable side-effects in metabolic engineering projects.

**Electronic supplementary material:**

The online version of this article (doi:10.1186/s12934-015-0381-7) contains supplementary material, which is available to authorized users.

## Background

Engineering of microorganisms for the production of isoprenoid compounds continues to attract intensive research efforts due to the high value and versatility of this class of natural products for industrial applications [1, 2]. Much progress has been made in recent years towards heterologous production of these sought-after molecules via microbial fermentation processes [3, 4]. However, fundamental questions about the regulation of the metabolic pathways leading to the production of isoprenoids are still unresolved, particularly in the methylerythritol-phosphate (MEP) pathway used by *Escherichia coli* [5, 6]. It is known that the ability to sustain heterologous isoprenoid production varies significantly between *E. coli* strains [7–10]. We speculate that understanding the molecular events that underpin this difference in capacity will, in turn, improve our understanding of the regulation of isoprenoid production.

Lycopene, a C_40_ carotenoid pigment, is widely used as a reporter of isoprenoid pathway flux. Due to its red color, differences in lycopene content can be estimated by visual inspection and quantified with a quick and inexpensive spectrophotometric method. This has led to lycopene becoming the metabolite of choice in high-throughput metabolic engineering studies of isoprenoid pathway flux [11–18]. Lycopene has been used to identify rate-limiting steps of the MEP pathway [7], test in silico predictions of metabolic fluxes in a pathway engineering context [16], and, more recently, to demonstrate the feasibility of improving a desired phenotype using multiplex automated genome engineering [11]. Importantly, several studies focused on identifying *E. coli* genes not directly involved in core MEP pathway reactions that affect lycopene production [12, 14, 19, 20]. Differential expression of these non-MEP pathway genes was used to increase lycopene production, often without an in-depth analysis of the mechanism leading to this phenotype. Implicitly, these association studies inferred a link between the identified overexpression- or knockout targets and changes in carbon flux towards lycopene.

Despite more than twenty non-MEP pathway genes having been associated with variation in lycopene production, it remains unclear whether changes in pigment accumulation are in fact related to flux through the isoprenoid pathway. The purpose of this study was to understand the effect of non-MEP pathway genetic factors on the production of lycopene in *E. coli*, and whether or not they influence MEP pathway flux.

In this study, a pair of genetically near-identical *E. coli* strains were identified that displayed a large difference in lycopene accumulation. By comparing *E. coli* with similar genetic backgrounds we aimed to understand the mechanisms behind differences in lycopene production and MEP pathway flux between these strains. Here we use genomics, quantitative proteomics and metabolomics as well as probing intracellular reactive oxygen species to demonstrate that lycopene production phenotypes in *E. coli* are not necessarily linked to changes in pathway flux.

## Results and discussion

### Lycopene production varies between wild-type and engineered *E. coli* strains

**Fig. 1:**
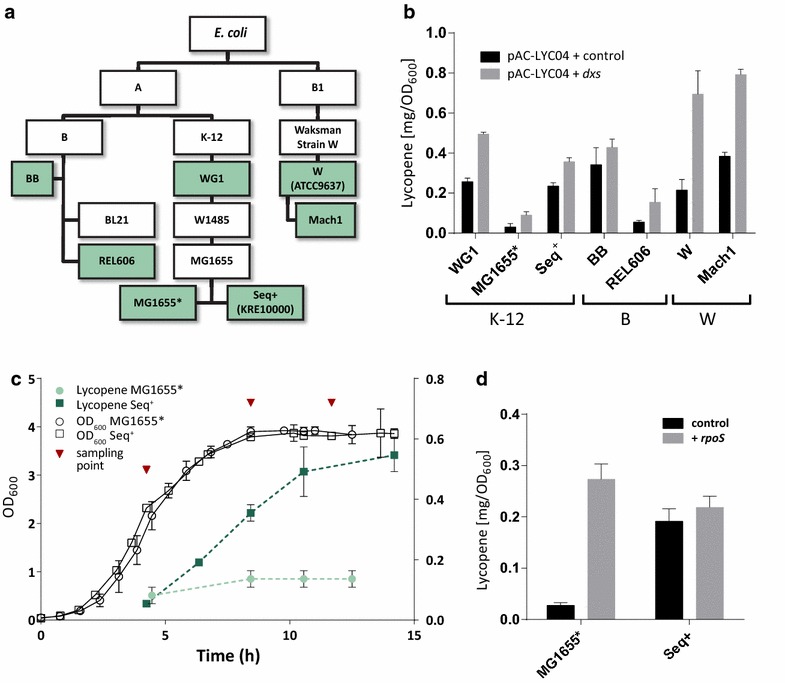
Inter-strain variability in lycopene accumulation and dependence on RpoS. **a** Relationships between *E. coli* strains used in this study. **b** Lycopene production in parental and derived laboratory strains from different phylogenetic groups. Production was measured either with the lycopene production plasmid (pAC-LYC04) plus empty vector plasmid (pTrc99SS), or with pAC-LYC04 and a *dxs* overexpression plasmid (pT-dxs). **c** Fermentation profile for lycopene production in a high (Seq^+^) and low (MG1655*) producer strain. Cells were grown in batch culture in 1 L bioreactors and samples for quantitative proteomics were collected in exponential, early and late stationary phase. **d** Functional complementation of the *rpoS* gene in the low producer MG1655* restores the lycopene production phenotype to high producer (Seq^+^) levels

Significant variability in lycopene production between common laboratory strains of *E. coli* has been reported previously [7, 21]. We first explored this variability across phylogenetic groups (A and B1), between isolates (B and K-12) and between parental strains and derivatives cured of phages and plasmids (e.g., WG1 versus MG1655*) (Fig. 1a).

Significant variation in the capacity to produce lycopene was observed between strains transformed with a lycopene production plasmid (Fig. 1b). A 12-fold difference was observed between the highest producer (Mach1, 0.38 ± 0.02 mg OD_600_^−1^) and the lowest producer (MG1655*, 0.03 ± 0.017 mg OD_600_^−1^). Large differences in lycopene production were also seen between closely related strains with the WG1 parental strain producing > sixfold more lycopene than its derivative, MG1655*. To test the hypothesis that growth rate affects lycopene production, a fast-growing derivative of MG1655, Seq^+^ (also described as KRE10000) [22–24], was included in the analysis. Seq^+^ differs from the reference MG1655 strain by a single nucleotide polymorphism (SNP) in the *rph* gene. This mutation affects expression of the downstream *pyrE* gene involved in pyrimidine biosynthesis, leading to a pyrimidine starvation phenotype and slower growth in *rph*^−^ strains such as MG1655. In Seq^+^ this mutation is repaired, therefore Seq^+^ has a slightly higher specific growth rate. The Seq^+^ strain produced lycopene at similar levels to the parental strain WG1 (Fig. 1b).

#### Variability in isoprenoid production under pathway engineering

Overexpression of deoxyxylulose-5-phosphate synthase (*dxs*) enhances production of lycopene (and several other isoprenoids, [6, 25]) in a variety of genetic backgrounds [14, 20, 26, 27], and Dxs is considered to be the rate-limiting enzyme for isoprenoid production in *E. coli* [7, 28]. In order to explore engineerability, all strains were co-transformed with a plasmid for overexpression of *dxs* (pT-dxs) as well as the lycopene production plasmid. All of the engineered strains accumulated more lycopene than their respective wild-types (Fig. 1b), but the degree to which lycopene production was increased by *dxs* overexpression differed greatly. The lowest increase in production was observed for *E. coli* BB (25 % increase ±9.8 %) and the highest was observed for *E. coli* W (323 % increase ±17 %).

The greater engineerability of W strains is of great practical interest. However, the large genetic dissimilarity between *E. coli* W and MG1655* [29], the best and worst lycopene producers (Fig. 1b), complicates a direct comparison between these two strains. Nevertheless, *E. coli* MG1655 is the most commonly used strain for isoprenoid production and its exceptionally low capacity to produce lycopene was noteworthy. We therefore focused on differences between strains in the K-12 lineage.

#### Complementation of *rpoS*, but not *rph*, increases lycopene accumulation

Genetic variability among different stocks of *E. coli* MG1655 is a recently recognized problem that can lead to inconsistent results between laboratories [30]. We therefore sequenced all K-12 lineage strains used in this study, identifying a number of previously described mutations common in MG1655-derivatives (Table 1). The only loss-of-function mutation that distinguished the low-producing MG1655* strain from the two high-producing K-12 strains, Seq^+^ and WG1, was the frame shift in the *rph* gene in MG1655*. When this mutation was repaired in an *rph*^−^ MAGE strain (Nuc5-.dnaG.Q576A.tolC-, a strain optimized for facile genome editing), a slight increase in growth rate was observed; however, lycopene production was unaffected (see Additional file 1).

**Table 1:** Genomic variation in the *E. coli* K-12 strains used in this study

Gene	Position	Type	Effect	MG1655^a^ reference	WG1^b^	MG1655*	Seq^+^
*rpoS*	2865477	SNP	STOP	C	T	T	C
*yedY*	2038457	SNP	Missense	C	A	A	C
*ycnA*	1335418	SNP	Missense	A	G	G	A
*yfcS*	1169059	SNP	Missense	T	C	C	T
*lamB*	4246613	Insertion	unknown	C	C	IS2	C
*crl*	257899	Insertion	Frame shift	IS1	No insertion	No insertion	IS1
*gatABC*	2171384	Deletion	Loss of operon	–	Deletion	Deletion	–
*glpR*	3558477	Deletion	Frame shift	–	No FS	No FS	–
*rph*	3813902	Insertion	Frame shift	–	G	–	G

^a^CGSC#: 7740, reference genome NC_000913.3

^b^CGSC#: 5073, not all identified mutations are listed here

It has previously been demonstrated that the stationary phase sigma factor RpoS (or σ^S^) and genes regulating its abundance positively affect lycopene production [14, 19, 20, 31]. Our MG1655* isolate contains a premature stop codon in the *rpoS* gene, a commonly occurring mutation in laboratory *E. coli* strains assumed to arise as an adaptation to typical laboratory growth conditions [32]. Seq^+^ accumulates sevenfold more lycopene than MG1655*, and does not have the *rpoS* amber mutation (Table 1). Complementation with a functional copy of *rpoS* under its native promoter restored lycopene production in MG1655* (Fig. 1d). The *rpoS* knockout was thus identified as the single factor responsible for the decreased lycopene production in MG1655*.

Interestingly, strain WG1 was one of the highest producers of the initial screen (Fig. 1b), and accumulates lycopene without a functional copy of *rpoS* (Table 1). Closer analysis of the WG1 genome, including the 34 other detected in-frame SNPs and INDELs, should provide insight into potential compensation mechanisms in this strain. One possibility is a partial rescue of the *rpoS*^+^ phenotype in WG1 through a missense mutation in the primary sigma factor *rpoD* (Y571H) that is not present in MG1655*, particularly since condition-dependent competition between these transcriptional regulators has been suggested recently [33]. Alternatively, WG1 might have other means to control cellular redox status (see below) that have been lost in MG1655*.

### The role of RpoS in lycopene production

While the importance of RpoS had been identified previously and confirmed in the above experiments, its mechanism of action in regulating lycopene production remained unclear. A detailed time-course comparison between an *rpoS*^+^ high lycopene producer (Seq^+^) and a low-producing Δ*rpoS* strain (MG1655*) was carried out in order to test whether RpoS controls isoprenoid biosynthesis through direct or indirect mechanisms. Direct control would imply that RpoS is responsible for increased concentration of MEP pathway or lycopene pathway enzymes in Seq^+^ compared to MG1655*. Transcriptomics studies published elsewhere suggest that MEP pathway transcription is not directly regulated by RpoS [33]. Therefore, RpoS might exert flux control indirectly by affecting cellular metabolism in a way that favors lycopene production, particularly since lycopene accumulation occurs mostly during stationary phase (Fig. 1c). For example, RpoS may be required for providing sufficient redox equivalents, maturation systems for MEP pathway enzymes, or removing competition for precursors from other pathways through protein degradation. These effects could not be examined using transcriptomics alone.

SWATH proteomics [34] was therefore used to quantify global differences in the proteome of *E. coli* MG1655* and Seq^+^ during lycopene production (Fig. 1c). Approximately 43 % of the total expressed proteome of *E. coli* [35] was quantified at three time points during the fermentation (1126 proteins with at least three peptides detected were quantified, from a total of 1759 proteins with at least one peptide detected). Due to the low abundance of MEP pathway proteins, only five of the eight pathway enzymes could be quantified using this approach; therefore, targeted selected reaction monitoring (SRM) methods were developed for quantification of all MEP and lycopene pathway enzymes.

#### Concentrations of MEP and lycopene pathway enzymes are not affected by RpoS

**Fig. 2:**
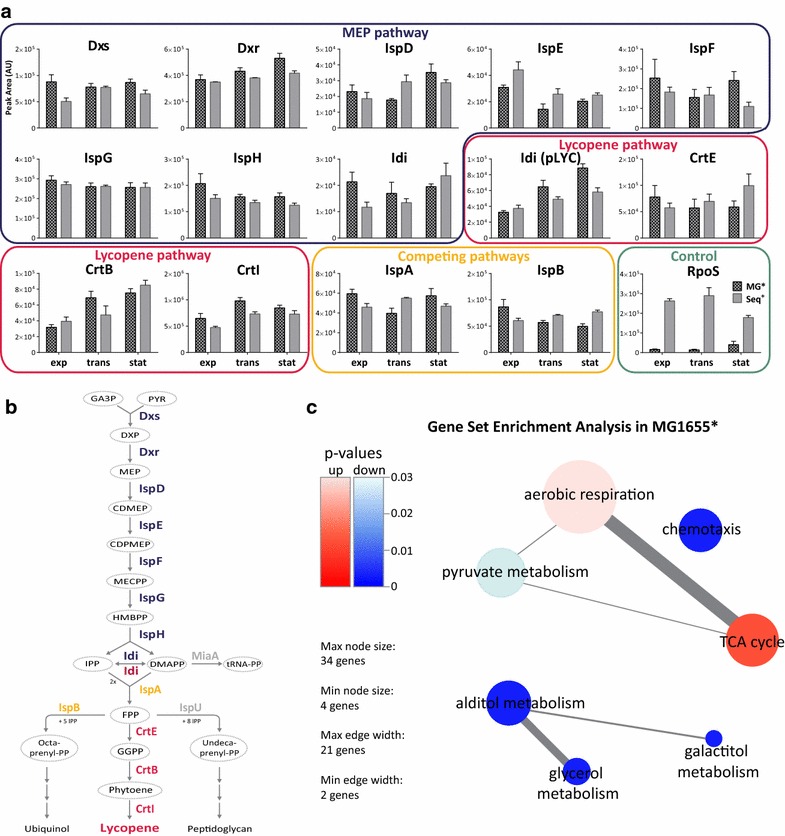
Quantitative proteomics comparison of Seq^+^ and MG1655* during lycopene production. **a** Selective Reaction Monitoring (SRM) proteomics for all MEP and lycopene production pathway enzymes measured in this study. **b** Lycopene biosynthesis pathway in *E. coli*. Shown are all enzymes involved in the formation of lycopene from glyceraldehyde-3-phosphate (GA3P) and pyruvate (PYR), as well as reactions competing for either isopentenyl pyrophosphate (IPP), dimethylallyl-pyrophosphate (DMAPP) or farnesyl-pyrophosphate (FPP). Peptides for MiaA and IspU were obtained but did not match our quality criteria and were excluded from this study. **c** Gene Set Enrichment Analysis of differentially expressed genes in the lycopene production phase. Up- or down-regulated proteins are shown for the Δ*rpoS* strain MG1655* relative to Seq^+^. Abbreviations: MEP pathway: Dxs, 1-deoxyxylulose-5-phosphate synthase; Dxr, 1-deoxy-d-xylulose-5-phosphate reductoisomerasease; IspD, 4-diphosphocytidyl-2-C-methyl-d-erythritol synthase; IspE, 4-diphosphocytidyl-2-C-methyl-d-erythritol kinase; IspF, 2-C-methyl-d-erythritol 2,4-cyclodiphosphate synthase; IspG, 1-hydroxy-2-methyl-2-(E)-butenyl 4-diphosphate synthase; IspH, hydroxymethylbutenyl diphosphate reductase; Idi, isopentenyl diphosphate isomerase. Competing pathways: IspA, farnesyl pyrophosphate synthase; IspB, octaprenyl diphosphate synthase; IspU, undecaprenyl diphosphate synthase; MiaA, tRNA(i^6^A37) synthase. pAC-LYC04: Idi(pLYC) from *H. pluvialis*; CrtE, geranylgeranyl diphosphate synthase from *P. agglomerans*; CrtB, phytoene synthase from *P. agglomerans*; CrtI, phytoene desaturase from *P. agglomerans*

All of the MEP pathway enzymes were present at similar levels in the *rpoS*^+^ (Seq^+^) and *rpoS*^−^ (MG1655*) strains (Fig. 2a, b). This is consistent with the lack of transcriptional effects published previously [33]. Expression of enzymes from the pAC-LYC04 plasmid was also comparable in the two strains, indicating that this was not the source of variation either. We could thus rule out the hypothesis that RpoS affects lycopene production through direct regulation of pathway capacity by means of influencing transcription, translation or protein degradation of MEP pathway or lycopene pathway enzymes.

#### Knockout of *rpoS* increases oxidative stress in stationary phase

The protein expression pattern was very similar during early and late stationary phase (Additional file 2) and data from these two time points were combined to enhance the statistical power and determine all differentially expressed proteins in the lycopene production phase. During lycopene production, 148 proteins were differentially expressed in the Δ*rpoS* strain MG1655* (false discovery rate < 0.05; see “Methods” for details on statistical analysis). Gene set enrichment analysis (GSEA) revealed that these can be grouped into five main categories: (1) chemotaxis, (2) galactitol metabolism, (3) C3 metabolism, particularly involving glycerol and pyruvate, (4) aerobic respiration and (5) TCA cycle (Fig. 2c) (Additional file 3). Induction of genes for chemotaxis and motility is known to be under complex regulatory control of alternative sigma factors including RpoS [36] and was assumed to have no effect on lycopene production in a bioreactor. The *gat* operon required for utilization of the alternative carbon source galactitol is deleted in MG1655* (Table 1). Proteins involved in galactitol metabolism were also assumed to have no significant effect in LB medium where catabolizable amino acids, not sugars, comprise the main carbon source [37] while galactitol is absent.

In contrast to the chemotaxis and galactitol proteins, the remaining GSEA groups—which are related to central carbon metabolism (CCM) and cellular redox state—present interesting targets for further examination. The concentration of proteins involved in C3 metabolism was decreased in MG1655*, whereas proteins involved in aerobic respiration and the TCA cycle were increased when compared to Seq^+^ (Fig. 2c). RpoS, the key regulator of the general stress response in *E. coli*, allows cells to adapt to various sub-optimal growth conditions—including lack of carbon source. As cells enter stationary phase, central carbon metabolism is rearranged and respiratory activity is reduced. The expression of TCA cycle and respiratory enzymes is down-regulated, presumably to prevent uncontrolled carbon draining and as a defense against accumulation of reactive oxygen species (ROS) produced during respiration (reviewed in [38]). Without RpoS, this mechanism is disabled in MG1655* and therefore TCA cycle and respiratory enzyme concentrations remain high in stationary phase.

**Fig. 3:**
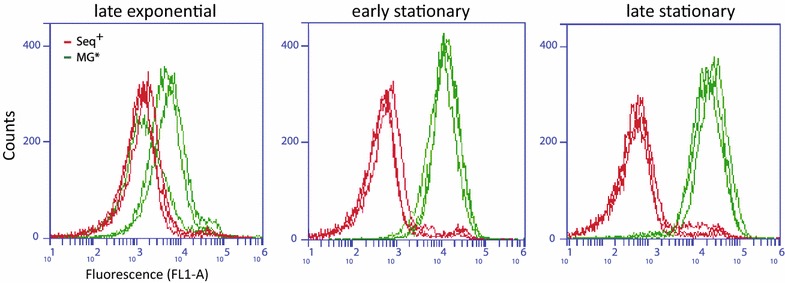
*rpoS* knockout causes oxidative stress in stationary phase. Cells were grown in the presence of the H_2_O_2_-activated fluorescent dye DHR123 and subjected to flow cytometry to measure intracellular ROS. *Peaks* represent distribution of fluorescence intensity from ten thousand cells per replicate (n = 3 replicates). The panels show DHR123-dependent fluorescence in MG1655* and Seq^+^ during exponential, early and late stationary growth phase, respectively

Significant differences were also observed in the concentration of individual stress-response proteins. The concentration of the stationary phase-specific catalase KatE (HPII), which is part of the RpoS sigmulon [39], was reduced >twofold in MG1655* (Additional file 3). On the other hand, a 1.5-fold increase was observed for the manganese-dependent superoxide dismutase, SodA. The expression of *sodA* is RpoS-independent, but it is induced in response to oxidative stress [40]. Taken together, these results suggested a compensatory response to elevated levels of reactive oxygen species (ROS) in MG1655*. The H_2_O_2_-activated fluorescent dye dihydrorhodamine 123 was therefore used to examine ROS levels during different growth phases. Indeed, a significant increase of intracellular H_2_O_2_ was detected in MG1655* compared to Seq^+^ during the transition from exponential to stationary phase growth (Fig. 3), confirming that higher concentrations of ROS accumulate in MG1655* in the absence of RpoS.

### Knockout of *rpoS* does not influence flux through the MEP pathway

**Fig. 4:**
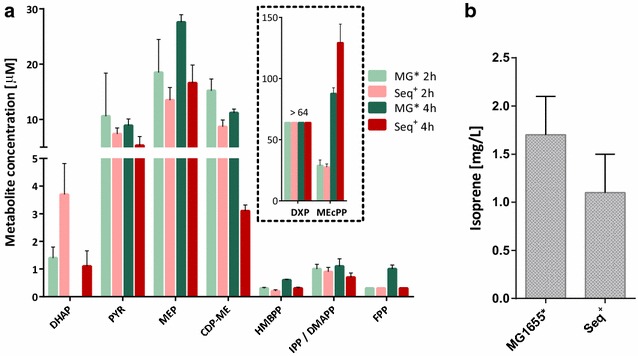
MEP pathway flux is not affected by RpoS. **a** Intracellular MEP pathway metabolite pools during lycopene production in MG1655* and Seq^+^ with *dxs* overexpression. Glyceraldehyde-3phosphate (GA3P), dihydroxyacetone-phosphate (DHAP), pyruvate (PYR), 1-deoxy-d-xylulose 5-phosphate (DXP), 2-C-methyl-d-erythritol-4-phosphate (MEP), 4-diphosphocytidyl-2-C-methylerythritol (CDP-ME), 2-C-methyl-d-erythritol-2,4-cyclodiphosphate (MEcPP), (E)-4-Hydroxy-3-methyl-but-2-enyl pyrophosphate (HMBPP), isopentenyl pyrophosphate (IPP)/dimethylallyl pyrophosphate (DMAPP; no chromatographic separation of IPP and DMAPP) and farnesyl pyrophosphate (FPP) were quantified using liquid chromatography tandem mass spectrometry (LC–MS/MS). 4-Diphosphocytidyl-2-C-methyl-d-erythritol-2-phosphate (CDP-MEP) was below detection level in all samples. **b** Isoprene production in the two strains harboring pT-ispS(L70R)

Despite the abundance of MEP and lycopene pathway enzymes being comparable in both strains, lycopene was barely detectable in MG1655*. Two of these enzymes, the iron sulphur cluster-containing proteins IspG and IspH are sensitive to oxygen levels [41]. Activity of these enzymes may therefore be decreased by high levels of oxidative stress. In the event of partial IspG or IspH inactivation, an accumulation of MEP pathway intermediates could be expected in MG1655*. In particular, methylerythritol cyclodiphosphate (MEcPP), the substrate for ispG, has been shown to accumulate to high concentrations under a range of conditions [42, 43]. However, analysis of MEP pathway metabolites during lycopene production showed that MEcPP did not accumulate in MG1655*; in fact, the intracellular concentration of MEcPP was slightly greater in Seq^+^ at 4 h post-induction (Fig. 4a). At this time point, the level of other intermediates both upstream (MEP and CDP-ME) and downstream (HMBPP, IPP/DMAPP and FPP) of MEcPP were higher in MG1655*, suggesting that—if anything—lycopene accumulation should be greater.

As an alternative measure of MEP pathway flux, isoprene production was examined in the two strains. Isoprene synthase uses DMAPP as a substrate, thus representing an immediate read-out of carbon flux through the MEP pathway. When a heterologous isoprene synthase was overexpressed, there was no significant difference in isoprene production between Seq^+^ and MG1655* (Fig. 4b). This result further confirmed that MEP pathway flux was unaffected by oxidative stress in MG1655* and indicated a lycopene-specific effect of the *rpoS* knockout.

### Oxidative stress decreases ‘measurable’ lycopene

Lycopene is a potent antioxidant, and is used by many plant species as a scavenger for reactive oxygen species (reviewed in [44]). The chromogenic conjugated double bonds of lycopene are readily oxidized, leading to loss of the characteristic red color and the formation of various colorless oxidation and cleavage products [45, 46]. The commonly used assay for lycopene quantification in *E. coli* is based on a spectrometric measurement of cell extracts at 472–475 nm, the lycopene-specific absorption maximum [7]. Therefore this assay quantifies only intact, fully conjugated lycopene molecules.

**Fig. 5:**
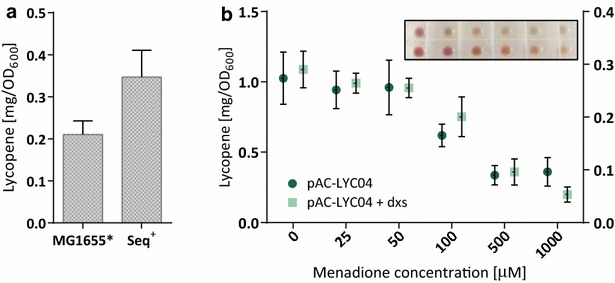
Excess reactive oxygen species decrease measurable lycopene content. **a** The stationary-phase specific catalase KatE was expressed under an arabinose-inducible promoter to complement *katE* expression in MG1655*. **b** Seq^+^ was grown in the presence of increasing concentrations of the redox cycling agent menadione. Values for Seq^+^ harboring pAC-LYC04 only, or in combination with pT-dxs, are shown on the *right* and *left axis*, respectively. The *inlet* shows cell pellets at the end of the fermentation with the increasing concentrations of menadione

We hypothesized that the apparent differences in lycopene production between Seq^+^ and MG1655* strains may in fact be due to lycopene degradation in the presence of increased levels of ROS in MG1655*. In stationary phase, when the majority of lycopene production occurs (Fig. 1c), the catalase KatE serves as the primary scavenger of H_2_O_2_. Expression of KatE is RpoS-dependent, and our proteomics results show that it was decreased twofold in MG1655* (Additional file 2). When grown on solid medium, overexpression of *katE* increased measurable lycopene in MG1655*, reducing the difference between Seq^+^ and MG1655* to less than two-fold from previously eightfold (Fig. 5a). This effect was not observed in liquid culture, suggesting further interplay between KatE and other factors in regulating oxidative stress under different growth conditions.

We next tested whether measurable lycopene content could be decreased by high levels of intracellular ROS. Therefore, the high lycopene producer Seq^+^ was treated with menadione, which induces ROS formation in *E. coli* [47, 48]. The measurable lycopene content decreased significantly in the presence of sublethal doses of menadione (Fig. 5b). Importantly, this phenotype was not a result of exogenous addition of ROS, but an effect of menadione-induced redox cycling leading to intracellular ROS generation [49], thereby resembling the situation in MG1655* (Fig. 3).

## Conclusions

We have carried out a systematic analysis of the variability in lycopene production phenotypes between different *E. coli* strains. We aimed to identify global regulators of flux through the MEP pathway in *E. coli* using lycopene as a read-out. It was found that lycopene production varies significantly in wild-type and engineered *E. coli* strains from different phylogenetic groups, and that strains respond differently to overexpression of the assumed primary rate-limiting enzyme Dxs. Our genomics analyses confirmed the previously described importance of RpoS for high-level lycopene accumulation [14, 20]; but we propose a new mechanism by which RpoS affects the lycopene production phenotype. RpoS has no effect on the abundance of any of the enzymes involved in lycopene biosynthesis. Furthermore, we demonstrate that knockout of *rpoS* does not affect the function of the iron-sulfur cluster proteins IspG or IspH, and in fact has no impact on MEP pathway flux under the conditions tested here. Instead, an excess of respiratory and TCA cycle activity combined with decreased expression of KatE (and possibly other ROS-scavenging enzymes) leads to oxidative stress in the Δ*rpoS* strain. We propose that this increase in ROS reduces the amount of measurable lycopene in producing *E. coli* cells by degrading the pigment to its colorless oxidation- and cleavage products.

These findings have significant implications for the study of lycopene production in *E. coli*. Firstly, it demonstrates that lycopene is not a suitable tool to study flux through isoprenoid pathways, for which it has been frequently used for nearly two decades [11–17]. Secondly, several studies that have identified genes ‘involved in lycopene biosynthesis’ should be carefully re-evaluated with regard to our findings. RpoS itself was identified independently at least twice in these shotgun approaches [14, 20], and several other genes involved in regulating either RpoS-dependent gene expression, degradation of RpoS, or stationary phase metabolism (*crl, iraD, iraM* and *appY*, respectively [13, 14, 20, 50]) were described to alter lycopene biosynthesis. While these genes may certainly be important for successful accumulation of intact lycopene (and potentially other oxygen-sensitive carotenoids) in *E. coli*, their role cannot be generalized to increasing flux towards lycopene or other isoprenoids. Some of the genes identified previously in associative studies most likely exert their effect on lycopene accumulation through a similar mechanism, and not through alteration of isoprenoid pathway flux. Therefore, we discourage the use lycopene and other carotenoids as reporter metabolites for isoprenoid production. If these molecules are used, it is strongly recommended to monitor cellular oxidative status after performing genetic modifications or when characterizing a new lycopene production phenotype. Instead, we recommend the use of isoprene as a reporter of MEP pathway flux since it combines a number of advantages: (1) isoprene is directly produced from DMAPP, one of two final metabolites of the MEP pathway [51], (2) only one heterologous enzyme (isoprene synthase) must be introduced, reducing the likelihood of unexpected side-effects [52, 53], (3) the high volatility of isoprene ensures that the product leaves cells efficiently [54, 55], (4) unlike lycopene, which sequesters into the cell membrane, isoprene has no known reactivity with *E. coli* metabolism or influence on cell growth. Isoprene has been widely used as a pathway reporter and industrial product in *E. coli* [56–59], the primary limitation being a lack of published inexpensive, high-throughput analysis systems.

Accessing the potential of the MEP pathway would theoretically provide a route to achieve higher yields of isoprenoids than we can currently achieve using the MVA pathway. We have presented a set of metabolomics and proteomics tools enabling us to analyze differences in flux through the MEP pathway, and thus assess the true capacity of a strain to direct carbon towards these high-value compounds. Finding a production host that responds well to metabolic engineering of this pathway may prove essential. While we have focused on the MEP pathway in the current study, our findings regarding the suitability of lycopene as an isoprenoid pathway flux reporter are equally applicable to the MVA pathway.

## Methods

### Chemicals and reagents

Lycopene (Cat. No. L9879), isoprene (Cat. No. I19551), dihydrorhodamine 123 (DHR, Cat. No. D1054), tributylamine (≥99.0 %, Cat. No. 90780), menadione sodium bisulfite (Cat. No. M5750), glyceraldehyde-3-phosphate (Cat. No. G5251), dihydroxyacetone-phosphate (Cat. No. D7137) and pyruvate (Cat. No. P2256) were purchased from Sigma-Aldrich (St. Louis, MO, USA). 1-Deoxy-d-xylulose 5-phosphate (DXP), 2-C-methyl-d-erythritol-4-phosphate (MEP), 4-diphosphocytidyl-2-C-methylerythritol (CDP-ME), 4-diphosphocytidyl-2-C-methyl-d-erythritol 2-phosphate (CDP-MEP, synthesis kit), 2-C-methyl-d-erythritol-2,4-cyclodiphosphate (MEcPP), (E)-4-Hydroxy-3-methyl-but-2-enyl pyrophosphate (HMBPP), isopentenyl pyrophosphate (IPP), dimethylallyl pyrophosphate (DMAPP) and farnesyl pyrophosphate (FPP) were purchased from Echelon Biosciences (Salt Lake City, UT, USA). Isopropyl-B-D-1-thiogalactopyranoside (IPTG, Cat. No. AST0487) was purchased from Astral Scientific (Taren Point, NSW, Australia). HPLC grade acetonitrile and acetic acid (AR Grade) were purchased from RCI Labscan (Bangkok, Thailand) and Labscan (Gliwice, Poland), respectively. Oligonucleotides were purchased from Integrated DNA Technologies (Singapore). Trypsin (Trypsin Gold, Mass Spectrometry Grade) was purchased from Promega (NSW, Australia).

### Gene, plasmid, and strain construction

All *E. coli* strains and plasmids used in this study are listed in Table 2. Reference numbers are provided where strains were obtained from the Coli Genetic Stock Center (CGSC), American Type Culture Collection (ATCC), or Addgene. All oligonucleotides used in this study are listed in Table 3.

**Table 2:** *Escherichia coli* strains and plasmids used in this study

Strain/plasmid	Relevant characteristics	References
*E. coli* WG1	E. coli K-12 wild type strain	CGSC#: 5073
*E. coli* MG1655	F-, λ-, *rph*-*1*	CGSC#: 7740
*E. coli* MG1655*	F-, *λ* ^−^, *rph*-*1, rpoS396(Am),* see Table 1	CGSC#: 7740/this study
*E. coli* MG-rph^+^	MG1655-derivative Nuc5^−^.dnaG.Q576A, *rph* ^+^	[60]/this study
*E. coli* Seq^+^ (KRE10000)	MG1655 *rph* ^+^	Obtained from Donald Court, [23, 75]
*E. coli* BB	*E.coli* B wild type strain	CGSC#: 2507
*E. coli* REL606	F-, *tsx*-*467 (Am)*, *araA230*, *lon*, *rpsL227 *(strR), *hsdR*-, *[mal* ^+^ *]*_*K*-*12*_ (λ^S^)	CGSC#: 12149
*E. coli* W	*E.coli* W wild type strain	ATCC: 9637 [29]
*E. coli* Mach1	Commercially available cloning host derived from *E. coli* W	Invitrogen™
*E. coli* Nuc5.dnaG.Q576A.tolC-	MG1655-derived MAGE strain used for markerless genome engineering	[76], Addgene strain #41699
pAC-LYC04	*cat*, *idi* from *H. pluvialis, crtEIB* from *P. agglomerans*	[77]
pTrc99SS	*bla*, P_trc_, truncated MCS (derived from pTrc99a)	[78]/this study
pT-dxs	*bla,* P_trc_, optimized *dxs* from *P. trichocarpa*	This study
pT-ispS (L70R)	*bla,* P_trc_, optimized *ispS* from *P. alba*	This study
pRpoS	*kan*, P_rpoS_, *rpoS* from MG1655 (derived from pET28a)	This study
pAra-KatE	*bla,* P_araBAD_, *katE* from *E. coli* MG1655 (derived from pKD46)	This study/[79]

The *rph*^−^ mutation was repaired in an MG1655-derived strain suitable for ssDNA recombineering, *E. coli* MG1655 ΔmutS::cat Δ(ybhB-bioAB)::[λcI857 N(cro-ea59)::tetR-bla] xonA-, recJ-,xseA-, exoX- and redα- dnaG.Q576A tolC- [60]. The strain was obtained from Addgene (https://www.addgene.org, strain #41699). Co-selection multiplex automated genome engineering (MAGE) was performed as described previously [61]. Briefly, 4 rounds of MAGE were performed using the oligonucleotide *rph* repair (Table 2) to repair the *rph*- mutation; co-selection for ampicillin sensitivity was performed using the ΔbioA::*bla* mut oligo described in [62]. Mutation of the *rph* locus was confirmed by Sanger sequencing (Table 3).

**Table 3:** Oligonucleotides used in this study

Name	Oligonucleotide sequence
*rph* repair	T*T*T*G*CCAGCGCCGCCTTCTGCGTCGCTACAATGGATTCGATTCCCCCTCGGGCCAGAGCCAACAAGATGAGTAGCTCTTCATGGGTGAAC
PEF-F02	ATAACAATTTCACACAGGAAACAGACCATGGGATTAAAGAGGAGAATACTAGATG
PEF-R02	CAAGCTTGCATGCCTGCAGGTCGACTCTAGATCAGGAACTCATGATTTCCAGTG
PEF-F04	ATAACAATTTCACACAGGAAACAGACCATGGGATTAAAGAGGAGAATACTAGATG
PEF-R04	CAAGCTTGCATGCCTGCAGGTCGACTCTAGATTAGCGTTCAAACGGCAGAAT
rpoS-fw	GGGTCGCGGATCCGAATTCATCACTGGCGGAAATGCCATTAC
rpoS-rev	TTGTCGACCATATGGGATCCTGCGTATGGGCGGTAATTTGAC
katE-fw	GCTCTAAGGAGGTTATAAAAAATGTCGCAACATAACGAAAAG
katE-rev	GAGGATGCGTCATCGCCATTTCAGGCAGGAATTTTGTCAATC

The sequence for the *Populus trichocarpadxs* gene was obtained from the published genome sequence (http://www.phytozome.net/poplar). Prediction of the chloroplast transit peptide was performed using the ChloroP 1.1 server (http://www.cbs.dtu.dk/services/ChloroP/). *E. coli* codon optimization and synthesis of this truncated (transit peptide removed) sequence was performed by Genscript (Piscataway, NJ, USA). The *dxs* sequence was amplified from the plasmid vector provided by Genscript using oligonucleotides PEF-F02 and PEF-R02 and sub-cloned at the University of Queensland Protein Expression Facility (St Lucia, QLD, Australia) into the pTispS(P.t.) plasmid, replacing the *ispS(P.t.)* gene. pTispS(P.t.) is pTrc99a plasmid harboring a truncated isoprene synthase gene from *Populus trichocarpa*, and was a gift from Professor Seon-Won Kim, Gyeongsang National University, Korea.

An isoprene synthase gene from *Populus alba* (Genbank accession number EF638224) [63] was truncated as described previously [51] to remove the transit peptide. In addition, an L70R mutation, which improves the specific activity of the enzyme [64] was introduced. *E. coli* codon optimization and synthesis of this truncated sequence was performed by Genscript. The plasmid pT-ispS(L70R) was generated by replacing the *ispS*(*P.t*.) gene in pTispS(P.t.) with the *ispS(L70R)* gene, using primers PEF-F04 and PEF-R04.

Plasmid pRpoS was generated by amplifying the *rpoS* gene, including its core promoter (686 bp upstream of the start codon), from *E. coli* MG1655 using the oligonucleotides rpoS-fw and rpoS-rev. Isothermal assembly [65] was performed to insert the P_rpoS_-*rpoS* construct into a pET28a backbone, replacing the T7 promoter and His-tag from the vector.

Plasmid pAra-katE was generated by amplifying the *katE* ORF from *E. coli* MG1655 and cloning it into the backbone of pKD46 [8] under control of the araBAD promoter. The lambda Red gene operon was replaced with the *katE* gene using isothermal assembly with the oligonucleotides katE-fw and katE-rev.

### Medium and growth conditions

All experiments described in this study were carried out with three biological replicates. All data are presented as mean ± standard deviation, unless stated otherwise.

#### Shake flask fermentations

All fermentations for lycopene production were performed in shake flasks unless stated otherwise. Strains were grown in Luria–Bertani (LB) medium [66] containing 34 μg chloramphenicol/ml to select for maintenance of the pAC-LYC04 plasmid. 100 µg ampicillin/ml was used to maintain plasmids pT-dxs or pAra-katE; and 50 µg kanamycin/ml for pRpoS. Pre-cultures were started from a single colony in 5 ml medium and were grown to stationary phase. A 1:100 dilution was used to inoculate 50 ml of fresh medium as the main culture. Cells were grown in unbaffled 250 ml flasks at 30 °C and 250 rpm orbital shaking.

#### Fermentation in bioreactors for proteomics sampling

For experiments in bioreactors, cells were cultivated as in “Shake flask fermentations”, but the 50 ml shake flask culture was grown to mid-exponential phase (OD_600_ ≈ 1) and then used to inoculate 200 ml of the same medium to a starting OD_600_ of 0.05. Cells were grown in a parallel bioreactor system (DasGip, Juelich, Germany) at 30 °C, pH was maintained at 6.9 by addition of 4 N NaOH, and dissolved oxygen (DO) was monitored by an external DO meter (Presens, Regensburg, Germany) and maintained at 80 % of air saturation. At an OD_600_ of 0.5, lycopene production was induced with 0.5 mM IPTG.

#### Lycopene production on solid media

For measurement of lycopene production after complementation with pAra-KatE, cells were transformed with the plasmid and grown O/N at 37 °C on LB agar plates containing 100 µg ampicillin/ml. They were incubated at room temperature for another 48 h before 3 colonies each were scraped from the plate, resuspended in 1 ml of H_2_O each and optical density and lycopene content were measured as described below.

### Analytics

#### Lycopene production assay

Production of lycopene was quantified as described previously [55]. Briefly, after measurement of the optical density (OD_600_), biomass from 1 ml of culture was extracted with 1 ml of acetone at the indicated time points. Lycopene absorption was measured at 475 nm and values were normalized by OD_600_.

#### Isoprene production

Isoprene production was measured as described previously [55]. Single colonies of *E. coli* Seq^+^ or *E. coli* MG1655* bearing pT-ispS(L70R) were used to inoculate 2 ml precultures in LB medium and grown at 37 °C until stationary phase was reached. Cells were diluted 1:100 into terrific broth (TB) with ampicillin, grown to an OD_600_ of 0.5, induced with 1 mM IPTG and grown for another 1.5 h at 30 °C. The culture was diluted 1:20 into 500 µl of fresh induction medium (TB [67] containing 100 µg ampicillin/ml and 1 mM IPTG) and grown in sealed 20 ml gas chromatography (GC) vials (Agilent Cat. No. 5188-2753 and 5188-2759) for 48 h at 30 °C. Isoprene production was quantified directly from the culture headspace as described in [55].

#### Metabolite measurements

Intracellular metabolites were quenched and extracted using a method adapted from [43]: At the indicated time points, the equivalent of 2 OD_600_ (*ml) of culture were centrifuged at 4 °C for 20 s at 16,000×*g*, the supernatant was discarded and the pellet snap-frozen in liquid nitrogen. The pellet was resuspended in 300 µl of 80 % MeOH (v/v) and metabolites were extracted by vortexing for 10 min at room temperature. Cell debris was removed by centrifugation at 4 °C for 15 min at 18,000×*g*. The samples were dried in a vacuum concentrator until the volume was <10 µl and then resuspended in ultrapure H_2_O to a final volume of 50 µl. One microliter of 0.5 mM AZT was added as an internal standard.

Liquid chromatography tandem mass spectrometry (LC–MS/MS) data were acquired on a Dionex UltiMate 3000 liquid chromatography system (Dionex, California, USA) coupled to an ABSciex 4000 QTRAP mass spectrometer (ABSciex, Concord, Canada). The liquid chromatography system was controlled by Chromeleon software (v6.80 SR9, Dionex), and chromatographic separation was achieved by injecting 10 μL onto a Gemini-NX C18 150 mm × 2 mm I.D., 3 μm 110 Å particle column (Phenomenex, Aschaffenburg, Germany) equipped with a pre-column Security Guard Gemini-NX C18 4 mm × 2 mm I.D. cartridge. The column oven temperature was controlled and maintained at 55 °C throughout the acquisition and the mobile phases (adapted from [68]) were as follows: 7.5 mM aqueous tributylamine adjusted to pH 4.95 (±0.05) with glacial acetic acid (eluent A) and acetonitrile (eluent B). The mobile phase flow rate was maintained at 300 μL/min throughout the following elution profile composed of eluent A and B: 100 % eluent A decreased to 85 % eluent A over 10 min, then decreased to 70 % eluent A over 25 min, then decreased to 30 % eluent A over 10 min, then decreased to 0 % eluent A over 2 min and held at 0 % eluent A for a further 2 min, then increased to 100 % over 1 min and held at 100 % A for 10 min. The gradient profile was introduced directly into the mass spectrometer with no split.

The mass spectrometer was controlled by Analyst 1.5.2 software (ABSciex) and was equipped with a TurboV electrospray source operated in negative ion mode. The following optimized parameters were used to acquire scheduled Multiple Reaction Monitoring (MRM) data: ionspray voltage −4500 V, nebulizer (GS1), auxiliary (GS2), curtain (CUR) and collision (CAD) gases were 60, 60, 20 and medium (arbitrary units), respectively. The auxiliary gas temperature was maintained at 350 °C. The analyte-dependent parameters for the detection of MEP pathway-related metabolites are given in Additional file 2. For all analytes the entrance potential (EP) was −10 volts.

The samples were run with sample- and analyte-relevant calibration standards and pooled QC samples [69, 70] to control for reproducibility of data acquisition and to ensure data integrity. Analyte stock solutions were prepared in purified water (Veolia) and aliquots of each solution were mixed to achieve a final calibrant solution at 50 μM. This calibrant solution was serially diluted and the dilutions used as calibration standards from 50 to 0.195 μM, constituting 5 ≤ *x* ≤ 9 calibration points to account for differential responses in the mass spectrometer. Data were processed using MultiQuant 2.1 software (ABSciex).

### Proteomics

#### SWATH and SRM sample preparation

Cell pellets for protein extraction were prepared in the same way as for metabolite extraction, see “Metabolite measurements”. Protein extraction and preparation for SWATH or targeted proteomics was performed using a method adapted from [71]: the frozen cell pellets were resuspended in 90 µl lysis buffer (50 mM Tris/HCl buffer, 1 mM EDTA, pH 7.5) containing 2 mg lysozyme/ml. Cell wall lysis was performed at 37 °C for 15 min. DNA and RNA were digested by incubation with DNase and RNase (2 U each) for an additional 10 min. For the solubilization of membrane proteins, 10 µl of 10 % SDS (w/v) was added and samples were incubated at 37 °C under vigorous shaking for 15 min. Cell debris was removed via centrifugation at 3345×*g* for 5 min and the supernatant was transferred and centrifuged at 11,300×*g* for 10 min. The crude protein extract was methanol/chloroform precipitated as described previously [72]. Protein precipitates were redissolved in 100 µl denaturation buffer containing 8 M urea in 10 mM Tris buffer. Protein concentration was measured against a BSA standard curve using the BCA assay (Pierce™ Cat. No. 23227, Thermo Fischer Scientific (VIC, Australia) according to the manufacturer’s instructions. Proteins were reduced with 1 mM dithiothreitol for 1 h at room temperature and then alkylated with 1 mM iodoacetamide (IAA) for 1 h at room temperature in the dark. Samples were diluted to <1 M urea with 900 µl 20 mM ammonium bicarbonate and digested with trypsin (1:50 w/w) O/N at 37 °C. Peptides were desalted using a Sep-Pak C18 cartridge (Waters, WAT054955) and eluted in 300 µl 70 % (v/v) acetonitrile, 0.1 % (v/v) trifluoric acid. Samples were dried in a vacuum concentrator and resuspended in 50 µl 0.1 % formic acid.

#### SWATH library reference and sample preparation

For SWATH peptide reference library generation, peptides from all biological samples (ca. 300 µg total) were pooled and separated using SCX chromatography-based fractionation as described previously [73]. Briefly, peptides were separated using a Zorbax 300-SCX column (5 µm, 4.6 × 50 mm) (Agilent) at 0.5 ml min^−1^ on an Agilent 1100 chromatography system. Fractions (250 µl) were collected in a microtitre plate and then pooled to give 12 fractions in total. C18 ZipTips (Millipore, Cat. No. ZTC18S096) were used for desalting. The SWATH reference library was generated from information-dependent acquisition (IDA) using the 12 fractions. The HRM Calibration Kit (Ki-3003) from Biognosys (Schlieren, Switzerland) was used as a retention time standard in all IDA and SWATH proteomics experiments. IDA and SWATH samples were injected twice (technical replicates) from 5 µg of peptides and from 0.5 µg of peptides, respectively.

#### LC–MS/MS analysis of SWATH samples

IDA and SWATH samples were separated on a Shimadzu Prominence nanoLC system as described previously [74], Briefly, peptides were separated on a Vydac Everest C18 (300 Å, 5 µm, 150 mm × 150 µm) column at a 1 µl/min flow rate, using a gradient of 10–60 % buffer B over 75 min, where buffer A = 1 % ACN/0.1 % FA and buffer B = 80 % ACN/0.1 % FA. Eluted peptides were directly analyzed on a Triple-TOF 5600 mass spectrometer (ABSciex) equipped with a Nanospray III interface. SWATH analyses were scanned across m/z 350–1800 for 0.05 s followed by high sensitivity DIA mode, using 26 Da (1 Da for window overlap) isolation windows for 0.1 s, across m/z 400–1250. Collision energy for SWATH samples was automatically assigned based on m/z mass windows by Analyst software.

#### SRM targeted proteomics

For method development, proteotypic peptides were synthesized for each of the following *E. coli* MG1655 proteins: Dxs, Dxr, IspD, IspE, IspF, IspG, IspH, Idi, IspA, IspB, IspU, MiaA, RpoS; as well as the proteins encoded on the pAC-LYC04 plasmid: Idi (*H.p*.), crtI (*P.a.*), CrtE (*P.a.*) and CrtB (*P.a.*). All peptides were synthesized by JPT (Berlin, Germany) as light SpikeTides™, dissolved according to manufacturer’s instructions and used to determine peptide retention times, dominant precursor and fragment ion masses. The top 5 fragment ions were selected for collision energy optimization. The three most intense fragment ions after optimization were used for the spectral library and later relative quantification of biological samples.

ESI based UPLC–MS/MS analyses were performed using a Shimadzu Prominence nano U-HPLC. Peptides were separated on an Aeris PEPTIDE XB-C18 Phenomenex column (1.7 μm 100 × 2.1 mm) at a 0.4 ml/min flow rate by a 36 min linear gradient of 5–50 % acetonitrile in H_2_O with 0.1 % formic acid (v/v). The UPLC was coupled with a triple quadrupole mass spectrometer equipped with a nano-LC electrospray ionization source (QTrap 5500, ABSciex, Forster City, CA, USA) that was operated in positive ion mode. For the validation process, the dwell time of each transition was set at 25 ms and a maximum of 50 transitions were scanned in one run. The resolution of Q1 and Q3 were both set at High and the entrance and collision cell exit potential were set at 10 and 30 V respectively. The ion spray voltage was set at 5500 V. Data acquisition was set to scheduled SRM, with a target window of 120 s and target scan time 1.5 s.

### Proteomics data analysis

#### SRM data analysis

Raw data were processed with Skyline v2.6 (http://proteome.gs.washington.edu/software/skyline/) and transition peak areas were exported after manual inspection and curation of the traces. Peptides were quantified by summing the integrated peak areas of three transitions. Protein abundance was quantified by summing the peptide abundances for that protein. Normalization for unequal amounts of protein loading was performed by selecting two housekeeping proteins (Pnp and TufB) from the SWATH dataset for which the abundance correlated strongly (r^2^ ≥ 0.945) with the sum of total integrated peak areas per sample. SRMs were developed for one peptide each and used to calculate a normalization factor that was applied to all other measured peak areas (Additional files 2, 4).

#### SWATH data bioinformatics analysis

MS/MS data from IDA and fractionated IDA were analyzed using the Paragon Algorithm from ProteinPilot v4.5 (ABSciex, Forster City, CA, USA). The 12 fractions from the fractionated IDA were analyzed simultaneously using ProteinPilot. Protein sequences for *E. coli* K-12 MG1655 were downloaded from NCBI Reference Sequence NC_000913.3 and Idi(Hp) and CrtE, CrtI and CrtB encoded on pAC-LYC04 were added to the .fasta file manually. Search parameters included trypsin digestion, IAA as cys-alkylation, FDR analysis and ‘thorough’ settings. Criteria for including proteins in the statistical analysis were identification of ≥3 peptides with ≥95 % confidence score and an unused score higher than the 5 % local false discovery rate score of all identified proteins. MS/MS data from SWATH was analyzed and quantified using PeakView v.1.2 (ABSciex, Forster City, CA, USA). Analysis was performed using the pooled sample (ProGroup output from ProteinPilot) as the spectra library for protein identification, containing 1126 proteins. A maximum of 5 peptides per protein and 10 transitions per peptide were used. Peptide modification was not allowed and shared peptides were excluded. Only peptides with 95 % confidence were used. An extraction window of 2 min was used.

#### Statistical analysis

SWATH protein output data from PeakView analysis was log2-transformed, quantile normalized and technical replicates were averaged. Statistical analysis of these data (three biological replicates per condition) was performed using the R (http://www.R-project.org) package ‘limma’ by fitting the data for each protein to a linear model, using a moderated t-statistic (adjusting results using empirical Bayes method) and adjusting for multiple testing (Benjamini-Hochberg). Proteins were classified as differentially expressed if the adjusted *p* value (Benjamini-Hochberg) was lower than 0.05.

#### Gene set enrichment analysis

Gene set enrichment analysis (GSEA) was performed using the ‘piano’ package in R. The list of gene level statistics generated with ‘limma’ was used as input for the runGSA function, using p-values and fold-changes for each gene. Annotation files for GO terms in *E. coli* K-12 were downloaded from http://www.geneontology.org. The network plot in Fig. 2b was generated using distinct-directional regulation (both directions) and a gene set p values ≤0.05. Alternative GSA methods were tested, but no other significantly enriched GO terms were found with p ≤ 0.05.

### ROS measurement

Flow cytometry analysis was performed using the H_2_O_2_-activated fluorescent dye dihydrorhodamine 123 (DHR123). Strains MG* and Seq^+^ (without plasmids) were grown in LB medium to stationary phase, then diluted to a starting OD_600_ of 0.1. At 2, 4 and 6 h after inoculation, 20 µl were transferred to a 96-well microtitre plate and DHR was added to a final concentration of 0.5 mM. After ca. 2 h cells were diluted 1:20 in phosphate-buffered saline (PBS) and fluorescence was immediately measured in an Accuri C6 flow cytometer (BD Biosciences). FL1-A fluorescence was measured for at least 10,000 events per sample with a flow rate of 14 µl/s, a threshold on FSC-H of 20,000 and no gating.

### Induction of intracellular ROS by menadione

*E. coli* strain Seq+ harboring pAC-LYC04 was grown in LB medium containing 34 μg chloramphenicol/ml; 100 µg ampicillin/ml was added for strains also harboring pT-dxs. Precultures were started from a single colony in 5 ml medium and were grown to stationary phase. A 1:100 dilution was used to inoculate 2 ml of fresh medium. Cells were grown in 24 deep-well plates (LifeTechnologies, cat no. CS15124) at 30 °C and 250 rpm orbital shaking. At an OD_600_ of 1–2, menadione was added at the indicated concentrations and cells were grown for 24 h before lycopene and final optical density were measured.

## Additional files

10.1186/s12934-015-0381-7 Lycopene production and specific growth rate in response to *rph* repair.
10.1186/s12934-015-0381-7 SWATH proteomics data. Contains a) PeakView output: raw data for ion, peptide and protein counts; b) output of statistical analysis: differentially expressed proteins during stationary phase.
10.1186/s12934-015-0381-7 SRM proteomics data. Contains Skyline output: raw and normalized data for transition, peptide and protein counts.
10.1186/s12934-015-0381-7 Additional LC-MS/MS acquisition parameters for the detection of MEP pathway-related metabolites.
