# Supplementary material for: Systems analysis of methylerythritol-phosphate pathway flux in E. coli: insights into the role of oxidative stress and the validity of lycopene as an isoprenoid reporter metabolite
Source: Microb Cell Fact. 2015 Nov 26;14:193. doi: 10.1186/s12934-015-0381-7 (PMC4662018; doi:10.1186/s12934-015-0381-7)
Supplement: Supplementary file 1 — 10.1186/s12934-015-0381-7 Lycopene production and specific growth rate in response to rph repair. [file 12934_2015_381_MOESM1_ESM.pdf]

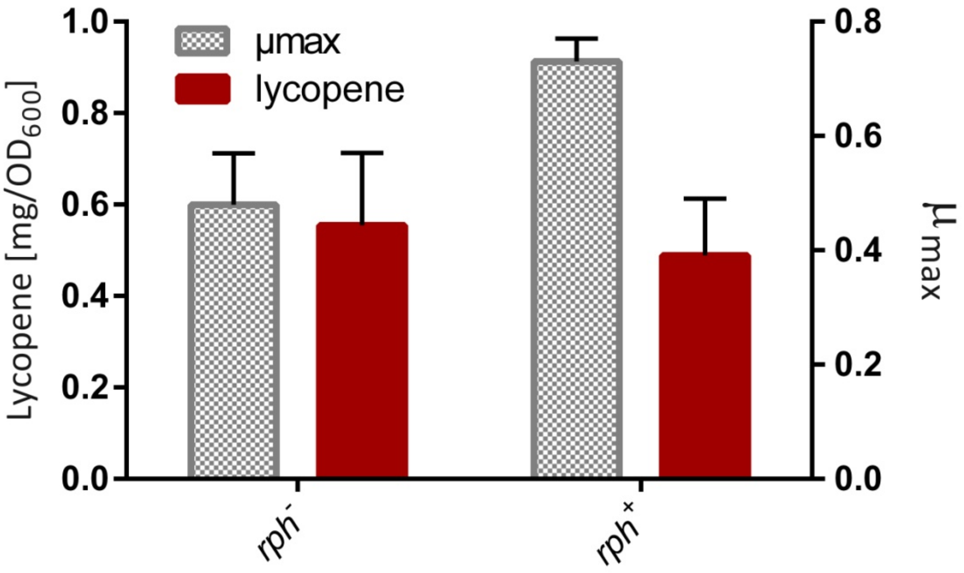


**Additional Figure 1: Lycopene production is not affected by *rph* repair.** The *rph*⁻ mutation was repaired using MAGE, and lycopene production (left axis) and specific growth rate (right axis) were compared.
