# Supplementary material for: Systems analysis of methylerythritol-phosphate pathway flux in E. coli: insights into the role of oxidative stress and the validity of lycopene as an isoprenoid reporter metabolite
Source: Microb Cell Fact. 2015 Nov 26;14:193. doi: 10.1186/s12934-015-0381-7 (PMC4662018; doi:10.1186/s12934-015-0381-7)
Supplement: Supplementary file 4 — 10.1186/s12934-015-0381-7 Additional LC-MS/MS acquisition parameters for the detection of MEP pathway-related metabolites. [file 12934_2015_381_MOESM4_ESM.docx]

Compound dependent parameters used in scheduled Multiple Reaction Mode

| Compound | Q1 Mass  (Da) | Q3 Mass  (Da) | RT  (Min) | DP  (V) | CE  (V) | CXP  (V) |
| --- | --- | --- | --- | --- | --- | --- |
| AZT1 | 265.8 | 42 | 7.2 | -70 | -34 | -1 |
| AZT2 | 265.8 | 193 | 7.2 | -70 | -20 | -11 |
| AZT3 | 265.8 | 223 | 7.2 | -70 | -16 | -1 |
| PYR | 87.02 | 43 | 6.6 | -45 | -12 | -1 |
| GA3P | 168.84 | 97.1 | 5.7 | -40 | -10 | -5 |
| DHAP | 168.84 | 97 | 7.3 | -50 | -14 | -5 |
| DXP_1 | 212.687 | 79.099 | 7.9 | -40 | -12 | -5 |
| DXP_2 | 212.687 | 96.9 | 7.9 | -40 | -18 | -5 |
| DXP_3 | 212.687 | 139 | 7.9 | -40 | -12 | -1 |
| MEP_1 | 214.718 | 62.989 | 6.5 | -55 | -120 | -7 |
| MEP_2 | 214.718 | 79.017 | 6.5 | -55 | -50 | -3 |
| MEP_3 | 214.718 | 96.986 | 6.5 | -55 | -26 | -7 |
| IPP_1 | 244.512 | 63 | 20 | -35 | -116 | -1 |
| IPP_2 | 244.512 | 79.011 | 20 | -35 | -38 | -5 |
| IPP_3 | 244.512 | 158.88 | 20 | -35 | -20 | -1 |
| DMAPP_1 | 244.983 | 63.035 | 20.5 | -35 | -118 | -1 |
| DMAPP_2 | 244.983 | 78.997 | 20.5 | -35 | -30 | -5 |
| DMAPP_3 | 244.983 | 158.919 | 20.5 | -35 | -18 | -9 |
| FPP_1 | 380.875 | 158.874 | 36.9 | -40 | -26 | -13 |
| FPP_2 | 380.875 | 63.012 | 36.9 | -40 | -130 | -1 |
| FPP_3 | 380.875 | 79.023 | 36.9 | -40 | -58 | -1 |
| CDP-ME_1 | 519.88 | 322.035 | 13.6 | -90 | -36 | -7 |
| CDP-ME_2 | 519.88 | 79.033 | 13.6 | -90 | -102 | -1 |
| CDP-ME_3 | 519.88 | 96.833 | 13.6 | -90 | -66 | -5 |
| CDP-MEP_1 | 599.909 | 277.103 | 23 | -140 | -34 | -9 |
| CDP-MEP_2 | 599.909 | 79.124 | 23 | -140 | -124 | -3 |
| CDP-MEP_3 | 599.909 | 159.121 | 23 | -140 | -76 | -5 |
| MECPP_1 | 276.737 | 78.509 | 15.2 | -125 | -56 | -1 |
| MECPP_2 | 276.737 | 96.964 | 15.2 | -125 | -34 | -1 |
| MECPP_3 | 276.737 | 179.077 | 15.2 | -125 | -30 | -3 |
| HMBPP_1 | 260.733 | 78.903 | 15.4 | -45 | -68 | -5 |
| HMBPP_2 | 260.733 | 158.764 | 15.4 | -45 | -26 | -11 |
| HMBPP_3 | 260.733 | 62.81 | 15.4 | -45 | -128 | -7 |
